# High Protein Diet Feeding Aggravates Hyperaminoacidemia in Mice Deficient in Proglucagon-Derived Peptides

**DOI:** 10.3390/nu14050975

**Published:** 2022-02-25

**Authors:** Shinji Ueno, Yusuke Seino, Shihomi Hidaka, Ryuya Maekawa, Yuko Takano, Michiyo Yamamoto, Mika Hori, Kana Yokota, Atsushi Masuda, Tatsuhito Himeno, Shin Tsunekawa, Hideki Kamiya, Jiro Nakamura, Hitoshi Kuwata, Haruki Fujisawa, Megumi Shibata, Takeshi Takayanagi, Yoshihisa Sugimura, Daisuke Yabe, Yoshitaka Hayashi, Atsushi Suzuki

**Affiliations:** 1 Departments of Endocrinology, Diabetes and Metabolism, Fujita Health University Graduate School of Medicine, Toyoake 470-1192, Japan; shinji0413jp@gmail.com (S.U.); sakai220@fujita-hu.ac.jp (S.H.); kana-y1215-ponyo@ezweb.ne.jp (K.Y.); drtommy88@gmail.com (A.M.); hfujisawa-spring@nifty.com (H.F.); megumi03@fujita-hu.ac.jp (M.S.); haratake@fujita-hu.ac.jp (T.T.); sugiyosi@fujita-hu.ac.jp (Y.S.); aslapin@fujita-hu.ac.jp (A.S.); 2 Department of Endocrinology, Hekinan City Hospital, Hekinan 447-8502, Japan; psychoplasma5@gmail.com; 3 Department of Clinical Oncology and Chemotherapy, Nagoya University Hospital, Nagoya 466-8550, Japan; y.takano@med.nagoya-u.ac.jp; 4 Department of Endocrinology, Research Institute of Environmental Medicine, Nagoya University, Nagoya 464-8601, Japan; yam@riem.nagoya-u.ac.jp (M.Y.); mihori@riem.nagoya-u.ac.jp (M.H.); 5 Department of Endocrinology, Nagoya University Graduate School of Medicine, Nagoya 466-8550, Japan; 6 Division of Diabetes, Department of Internal Medicine, Aichi Medical University School of Medicine, Nagakute 480-1195, Japan; thimeno@aichi-med-u.ac.jp (T.H.); tsune87@aichi-med-u.ac.jp (S.T.); hkamiya@aichi-med-u.ac.jp (H.K.); jiro@aichi-med-u.ac.jp (J.N.); 7 The Division of Diabetes, Clinical Nutrition and Endocrinology, Kansai Electric Power Hospital, Osaka 553-0003, Japan; hitoshi.kuwa@gmail.com; 8 Yutaka Seino Distinguished Center for Diabetes Research, Kansai Electric Power Medical Research Institute, Kobe 650-0047, Japan; ydaisuke@gifu-u.ac.jp; 9 Department of Diabetes, Endocrinology and Metabolism, Gifu University Graduate School of Medicine, Gifu 501-1194, Japan; 10 Department of Rheumatology and Clinical Immunology, Gifu University Graduate School of Medicine, Gifu 501-1194, Japan; 11 Center for Healthcare Information Technology, Tokai National Higher Education and Research System, Nagoya 464-8601, Japan; 12 Division of Molecular and Metabolic Medicine, Kobe University Graduate School of Medicine, Kobe 650-0017, Japan

**Keywords:** protein diet, glucagon, hyperaminoacidemia, food intake, hypoglycemia

## Abstract

(1) Background: Protein stimulates the secretion of glucagon (GCG), which can affect glucose metabolism. This study aimed to analyze the metabolic effect of a high-protein diet (HPD) in the presence or absence of proglucagon-derived peptides, including GCG and GLP-1. (2) Methods: The response to HPD feeding for 7 days was analyzed in mice deficient in proglucagon-derived peptides (GCGKO). (3) Results: In both control and GCGKO mice, food intake and body weight decreased with HPD and intestinal expression of *Pepck* increased. HPD also decreased plasma FGF21 levels, regardless of the presence of proglucagon-derived peptides. In control mice, HPD increased the hepatic expression of enzymes involved in amino acid metabolism without the elevation of plasma amino acid levels, except branched-chain amino acids. On the other hand, HPD-induced changes in the hepatic gene expression were attenuated in GCGKO mice, resulting in marked hyperaminoacidemia with lower blood glucose levels; the plasma concentration of glutamine exceeded that of glucose in HPD-fed GCGKO mice. (4) Conclusions: Increased plasma amino acid levels are a common feature in animal models with blocked GCG activity, and our results underscore that GCG plays essential roles in the homeostasis of amino acid metabolism in response to altered protein intake.

## 1. Introduction

Amino acids serve not only as building blocks for proteins but also as substrates for gluconeogenesis. Carbohydrate intake is reduced in a high-protein diet (HPD), which has been shown to have beneficial effects on metabolic control in patients with type 2 diabetes mellitus (T2DM) [1,2]. HPD is also considered beneficial for increasing protein synthesis and preventing sarcopenia in the elderly [3,4,5]. In addition to these beneficial effects, HPD has been shown to stimulate the secretion of glucagon (GCG) [6,7,8,9,10,11,12], which potently increases blood glucose levels in T2DM patients [6,13,14,15].

GCG plays pivotal roles in regulating the metabolism of not only glucose but also amino acids [16]. In addition, multiple peptides, including glucagon-like peptide-1 (GLP-1) and glucagon-like peptide-2 (GLP-2) are produced from their common precursor, proglucagon, which is encoded by the GCG gene (*Gcg*). Various animal models with defects in GCG activity have been established [17,18,19,20,21]. These models include mice deficient in the GCG receptor (GcgRKO), mice administered with blocking antibodies for GCG receptors [17], and mice with *Gcg* modifications [20]. The *Gcg* knockout (GCGKO) mice utilized in this report are homozygous for a *Gcg*-green fluorescent protein knock-in allele and lack all peptides derived from proglucagon, including GCG and GLP-1 [20,21]. While GcgRKO mice display lower blood glucose levels, GCGKO mice are euglycemic. These discrepancies have been attributed to the presence or absence of GLP-1 activity in GcgRKO or GCGKO mice, respectively. On the other hand, Increased plasma amino acid levels, such as glutamine, glycine, lysine, and especially alanine, have been documented in GcgRKO mice, GCGKO mice, mice treated with glucagon receptor antibody, and mice with diphtheria toxin mediated-ablation of alpha and L cells (Gluc-DTR), due to decreased expression levels of genes involved in amino acid catabolism [21,22,23,24,25,26]. These results indicate that GLP-1 plays a relatively minor role in the control of amino acid metabolism. In addition, the increased plasma amino acid levels are apparent after acute intraperitoneal administration of mixed amino acids in mice treated with glucagon receptor antibody and Gluc-DTR mice [26]. Nevertheless, the role of proglucagon-derived peptides, especially GCG, in the whole-body response to changes in oral protein intake has only been partially elucidated.

GLP-1 and glucose-dependent insulinotropic polypeptide (GIP) are the major incretins that stimulate insulin secretion in response to food ingestion. GLP-1 and GIP have multiple functions, such as control of gastrointestinal movement and satiety. Recently, it was reported that plasma GCG levels are increased, whereas those of GIP and insulin are decreased, in patients with T2DM on a HPD [9,10,27]. Therefore, the interplay of incretins, GCG, and insulin likely plays an important role in the control of metabolism in response to increased protein intake. However, such a metabolic response has been only partially characterized thus far. This study aimed to analyze the metabolic effect of HPD feeding in the presence or absence of proglucagon-derived peptides, including GCG and GLP-1.

## 2. Materials and Methods

### 2.1. Experimental Animals and Diets

Eight-week-old male C57BL/6J wild-type (WT) mice were obtained from CLEA Japan (Osaka, Japan). GCGKO mice on a C57BL/6 background were established and maintained [20]. Glucagon and GLP-1 are produced from the same precursor proglucagon. To generate GCGKO mice, a targeting vector was constructed in which the sequence that corresponded to exon 2, intron 2, exon 3, and part of intron 3 of proglucagon was replaced by the EGFP cDNA sequence, a polyadenylation signal, and a PGK promoter-driven G418 resistance cassette [20]. The absence of mRNA encoding proglucagon in the GCGKO mice has been extensively analyzed previously; hence, the GCGKO mouse is considered a “null mutant”, lacking all the proglucagon-derived peptides [20]. GCGKO male mice and *Gcg* heterozygous female mice were intercrossed to obtain male GCGKO and *Gcg* heterozygous mice, which served as controls, as previously described [21,28,29]. The mice were housed under a standard 12:12 h light:dark cycle, with free access to food and water. WT, *Gcg* heterozygous, and GCGKO mice were divided into two groups: mice fed normal chow (NC) (TestDiet^®^ #5755 with carbohydrates at 59.6%, protein at 18.3%, and fat at 22.1% of total energy; Japan SLC, Hamamatsu, Japan) and HPD (TestDiet^®^ #5787, with carbohydrates at 15.3%, protein at 61.3%, and fat at 23.4% of total energy; Japan SLC). All animal experimental procedures were carried out according to a protocol approved by the Institutional Animal Care and Use Committee of the Nagoya or Fujita Health University.

### 2.2. Plasma Biochemical Analyses

Blood was collected from the tip of the tails, and blood glucose levels were measured with an Antsense Duo Small Electrode Glucose Analyzer (Horiba, Kyoto, Japan). Blood samples were centrifuged (13,500 rpm, 10 min, 4 °C) twice, and the collected plasma samples were stored at −80 °C until the biochemical analyses. Plasma hormone levels were measured using the following assays: insulin, Mouse/Rat Insulin ELISA Kit (Morinaga Institute of Biological Science, Kanagawa, Japan); FGF21, Mouse and Rat FGF21 ELISA Kit (BioVendor Inc., Brno, Czech Republic); GCG, Glucagon ELISA Kit (Mercodia, Uppsala, Sweden); active GLP-1, GLP-1 Active Form Assay Kit (IBL, Gunma, Japan); GIP, GIP (total) ELISA kit (Millipore, Billerica, MA, USA); and corticosterone, Corticosterone Enzyme Immunoassay Kit (Arbor Assays, Ann Arbor, MI, USA). Plasma amino acid concentrations were measured by standard assays at SRL, Tokyo, Japan [21].

### 2.3. Isolation of RNA and Quantitative PCR

Mice were sacrificed after feeding. Liver, brown adipose tissue (BAT), white adipose tissue (WAT), and skeletal muscles (tibialis anterior, gastrocnemius, and quadriceps femoris) were collected. The duodenum was sampled from its proximal end, and the jejunum was sampled from the middle part of the proximal duodenum and distal ileum [30]. Extraction of RNA, synthesis of cDNA, and PCR (qPCR) were performed as previously reported [31,32]. Briefly, total RNA was reverse-transcribed using the ReverTra Ace quantitative qPCR RT Master Mix (TOYOBO, Osaka, Japan). After cDNA synthesis, qPCR was carried out in a 25 μL reaction containing THUNDERBIRD SYBR qPCR Mix (TOYOBO) using the ABI PRISM 7900HT Sequence Detection System (Applied BioSystems, MA, USA). The primer sequences are shown in Table 1. The mRNA levels of the genes of interest were normalized by those of β-actin and expressed relative to that of control mice fed with NC.

### 2.4. Measurement of Food Consumption

Each mouse was housed in an individual cage for 3 days for acclimatization, after which food consumption over 24 h was measured as previously reported [33]. Measurement of food intake was performed as an independent experiment.

### 2.5. Measurement of Liver Triglycerides and Glycogen Content

To determine the liver triglyceride content, 40–50 mg of each liver sample was homogenized using a Powermasher with 500 µL isopropanol. After centrifugation (2000× *g*, 15 min), 4 µL of the supernatant was analyzed using Triglyceride E test (FUJIFILM Wako Pure Chemical Co., Osaka, Japan) [34]. To determine the liver glycogen level, 10 mg of each liver sample was rapidly homogenized with 200 µL ddH_2_O on ice and was then boiled for 10 min to inactivate the enzyme. After centrifugation (18,000 rpm, 10 min), the supernatant was analyzed using a Glycogen Colorimetric Assay Kit II (BioVision, Milpitas, CA, USA).

### 2.6. Insulin Tolerance Test

Mice were deprived of food for 6 h before the administration of an insulin tolerance test (ITT). Insulin was injected intraperitoneally at a dose of 0.75 U/kg. Blood glucose levels were measured 0, 30, 60, 90, and 120 min after insulin injection. ITT was performed as an independent experiment.

### 2.7. Statistical Analysis

Results are expressed as mean ± SEM. Statistical analysis was evaluated using unpaired Student’s *t*-test or one-way ANOVA. Differences between groups were considered statistically significant when *p* values were <0.05. GraphPad Prism 9 for Windows (GraphPad Software, San Diego, CA, USA) was used for statistical analysis.

## 3. Results

### 3.1. Hormonal Responses to HPD in WT Mice

**Figure 1:**
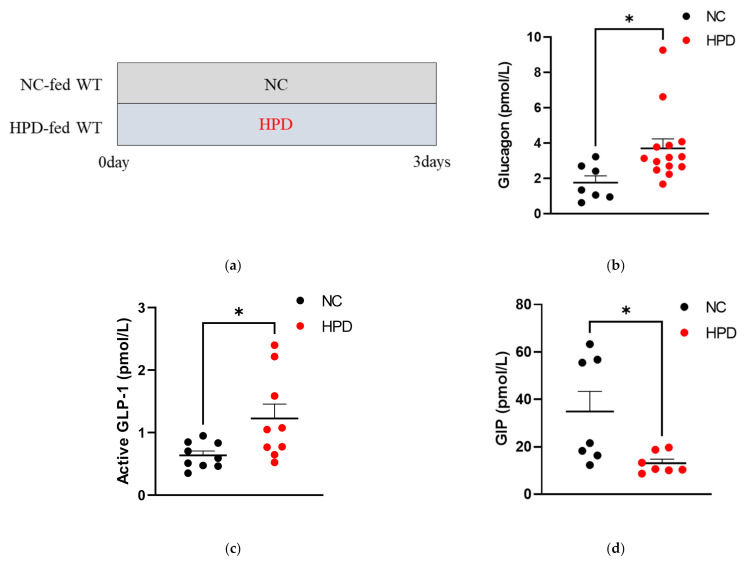

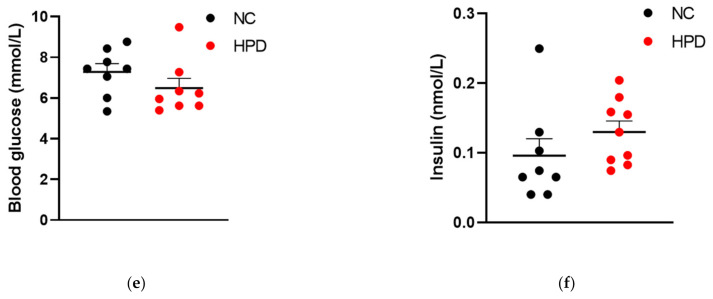
Hormonal responses to high-protein diet (HPD) in wild-type (WT) mice. (**a**) Study design. NC group: mice fed NC for 3 days; HPD group: mice fed ST for 3 days. Plasma concentrations of (**b**) glucagon (GCG), (**c**) active GLP-1, (**d**) total GIP, (**e**) glucose, and (**f**) insulin levels in WT mice fed normal chow (NC, black dots; *n* = 7–9) and HPD (red dots; *n* = 7–14) under ad libitum feeding conditions. Plasma samples were collected 3 days after beginning the diet (* *p* < 0.05, relative to NC). Data are expressed as mean ± SEM.

To analyze hormonal responses to HPD feeding, we fed WT C57BL/6J mice with HPD or NC for 3 days and examined the plasma concentration of pancreatic/gastrointestinal hormones under ad libitum feeding conditions. Plasma GCG (Figure 1b) and active GLP-1 (Figure 1c) levels were significantly higher in mice fed HPD than those fed NC, whereas plasma GIP levels (Figure 1d) were significantly lower in mice fed HPD. No significant difference in blood glucose levels was observed (Figure 1e). Plasma insulin levels were not significantly different between the two groups (Figure 1f).

### 3.2. HPD-Induced Changes in Body Weight, Food Intake, Gene Expression in BAT, and Plasma Awmino Acid Levels in WT Mice

**Figure 2:**
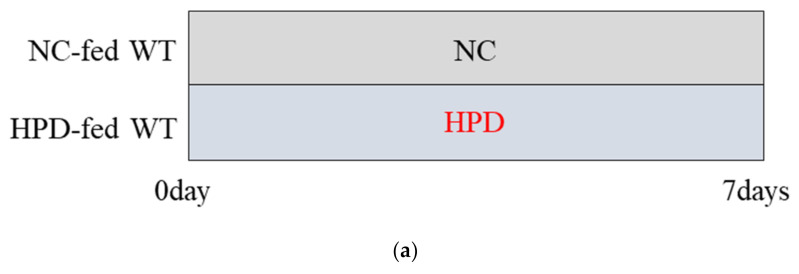

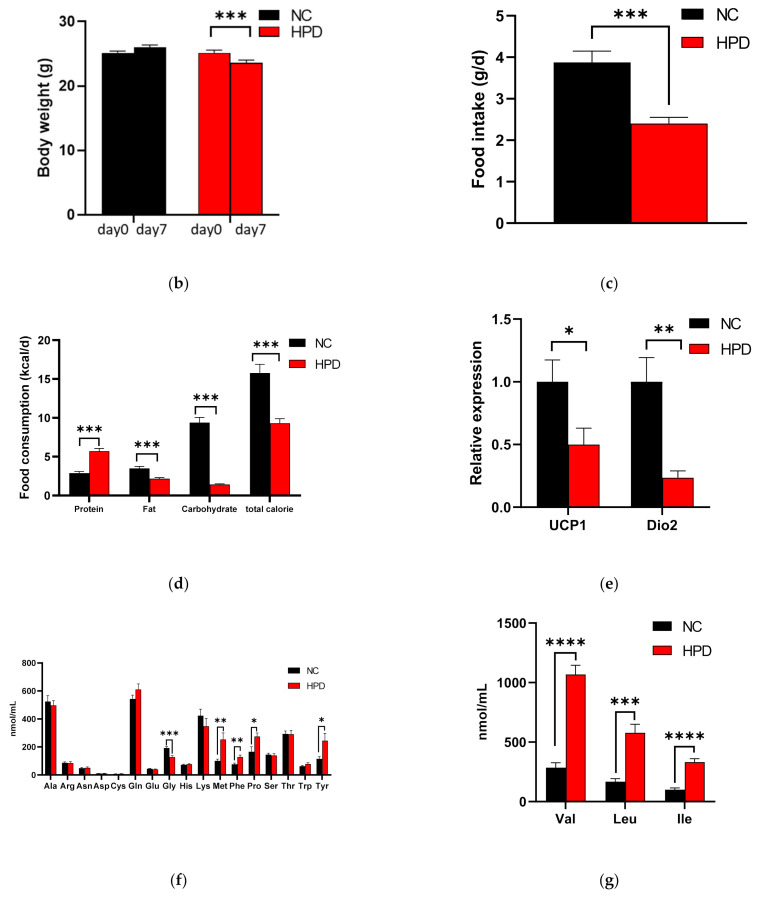
HPD-induced changes in body weight, food intake, gene expression in brown adipose tissue (BAT), and plasma amino acid levels in WT mice. (**a**) Study design. NC group: WT mice fed NC for 1 week; HPD group: WT mice fed HPD for 1 week. (**b**) Body weight, (**c**) food intake, (**d**) food consumption of protein, fat, carbohydrates, and total calories in WT mice fed NC (black bars; *n* = 8) and HPD (red bars; *n* = 8) after 1 week of feeding (*** *p* < 0.001, relative to NC). (**e**) mRNA expression levels of UCP1 and DIO2 in BAT in WT mice fed HPD (red bars; *n* = 8) relative to those in mice fed NC (black bars; *n* = 8) after 1 week of feeding (* *p* < 0.05, ** *p* < 0.01, relative to NC). Plasma (**f**) amino acid and (**g**) BCAA levels in WT mice fed NC (black bars; *n* = 8) and HPD (red bars; *n* = 8) after 1 week of feeding (* *p* < 0.05, ** *p* < 0.01, *** *p* < 0.005, **** *p* < 0.001, relative to NC). Data are expressed as mean ± SEM.

HPD feeding for 7 days resulted in a significant decrease in body weight (Figure 2b). Food consumption (Figure 2c) and caloric intake (Figure 2d) were significantly lower in HPD-fed mice. Accordingly, intake of carbohydrates and fat was also lower in HPD-fed mice. However, protein intake was significantly higher in HPD-fed mice and was approximately twofold that of NC-fed mice (Figure 2d). We also analyzed the expression levels of uncoupling protein 1 (*Ucp1)* and deiodinase iodothyronine type II (*Dio2*) mRNA in the BAT as surrogate markers for energy expenditure (Figure 2e). Expression of *Ucp1* and *Dio2* were significantly decreased in HPD-fed mice, suggesting that the contribution of energy expenditure to the body weight decrease was negligible compared to that of the decrease in caloric intake.

The plasma concentrations of 12 amino acids, including glutamine and alanine, were not different between HPD- and NC-fed mice, whereas that of glycine was significantly decreased in HPD-fed mice (Figure 2f). On the other hand, branched-chain amino acids were markedly increased in HPD-fed mice (Figure 2g). The concentrations of methionine, phenylalanine, proline, and tyrosine were also significantly increased in HPD-fed mice (Figure 2f).

### 3.3. HPD-Induced Changes in Food Intake and Contribution of Proglucagon-Derived Peptides

**Figure 3:**
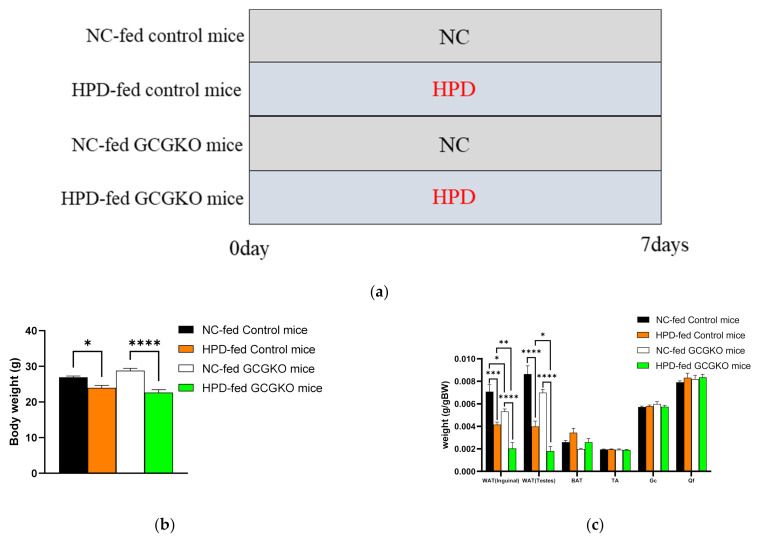

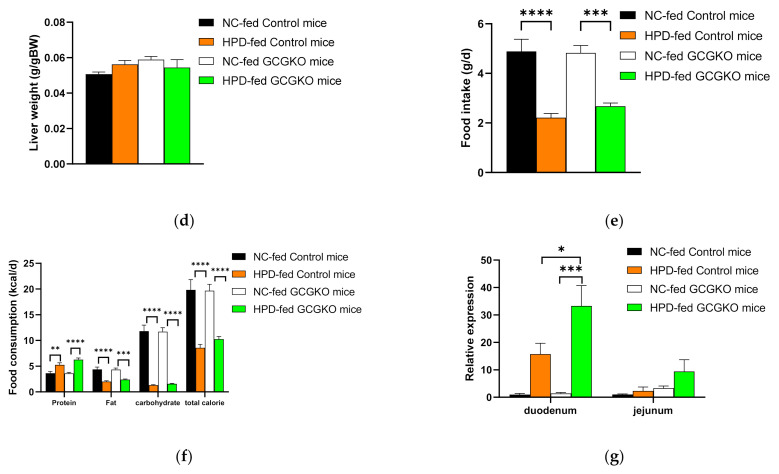
HPD-induced changes in food intake and contribution of proglucagon-derived peptides. (**a**) Study design. NC group: control and GCGKO mice fed NC for 1 week; HPD group: control and GCGKO mice fed HPD for 1 week. (**b**) Body weight, (**c**) WAT, BAT, skeletal muscle (TA: tibialis anterior; Gc: gastrocnemius; Qf: quadriceps femoris), and (**d**) liver weight in NC-fed control mice (black bars; *n* = 8), HPD-fed control mice (orange bars; *n* = 8), NC-fed GCGKO mice (white bars; *n* = 8), and HPD-fed GCGKO mice (green bars; *n* = 8) after 1 week of feeding (* *p* < 0.05, ** *p* < 0.01, *** *p* < 0.001, **** *p* < 0.0001). (**e**) Food intake and (**f**) food consumption of protein, fat, carbohydrates, and total calories in NC-fed control mice (black bars; *n* = 8), HPD-fed control mice (orange bars; *n* = 8), NC-fed GCGKO mice (white bars; *n* = 8), and HPD-fed GCGKO mice (green bars; *n* = 8) after 1 week of feeding (** *p* < 0.01, *** *p* < 0.001, **** *p* < 0.0001). (**g**) mRNA expression levels of Pepck in the duodenum and jejunum in NC-fed control mice (black bars; *n* = 6), HPD-fed control mice (orange bars; *n* = 8), NC-fed GCGKO mice (white bars; *n* = 7), and HPD-fed GCGKO mice (green bars; *n* = 8) after 1 week of feeding (* *p* < 0.05, *** *p* < 0.001). Data are expressed as mean ± SEM.

Body weight decreased after HPD feeding for 7 days in both the control and GCGKO mice, which lack all proglucagon-derived peptides, including GCG and GLP-1 (Figure 3b). Both GCG and GLP-1 exert static effects on gastrointestinal movement, and GLP-1 is a satiety hormone. However, food intake was suppressed to a similar extent in the GCGKO mice as the controls, suggesting that involvement of these peptides in HPD-induced suppression of food intake is limited (Figure 3e). The weight of the WAT was markedly decreased by HPD in both the control and GCGKO mice, whereas no significant change was observed in the weight of the BAT and skeletal muscles (Figure 3c). The weight of the WAT in HPD-fed GCGKO mice was significantly less than that in HPD-fed controls. On the other hand, no significant difference in liver weight was observed among the four groups (Figure 3d). Protein consumption was significantly increased in HPD-fed mice compared to NC-fed mice, whereas the intake of fat, carbohydrates, and total calories was significantly decreased (Figure 3f).

### 3.4. HPD Increased the Expression Levels of Phosphoenolpyruvate Carboxykinase (Pepck) mRNA in the Intestinal Tract in a GCG-Independent Manner

A protein-enriched diet is reported to induce intestinal gluconeogenesis and suppress food intake in rats [35]. Therefore, we analyzed *Pepck* expression, a marker for gluconeogenesis, in the duodenum and jejunum. The expression levels of *Pepck* mRNA in the duodenum were significantly increased by HPD feeding in the GCGKO mice, and similar data were observed in the control mice (Figure 3g).

### 3.5. Blood Glucose Levels and Hormonal Response to HPD Feeding in the Presence and Absence of Proglucagon-Derived Peptides

**Figure 4:**
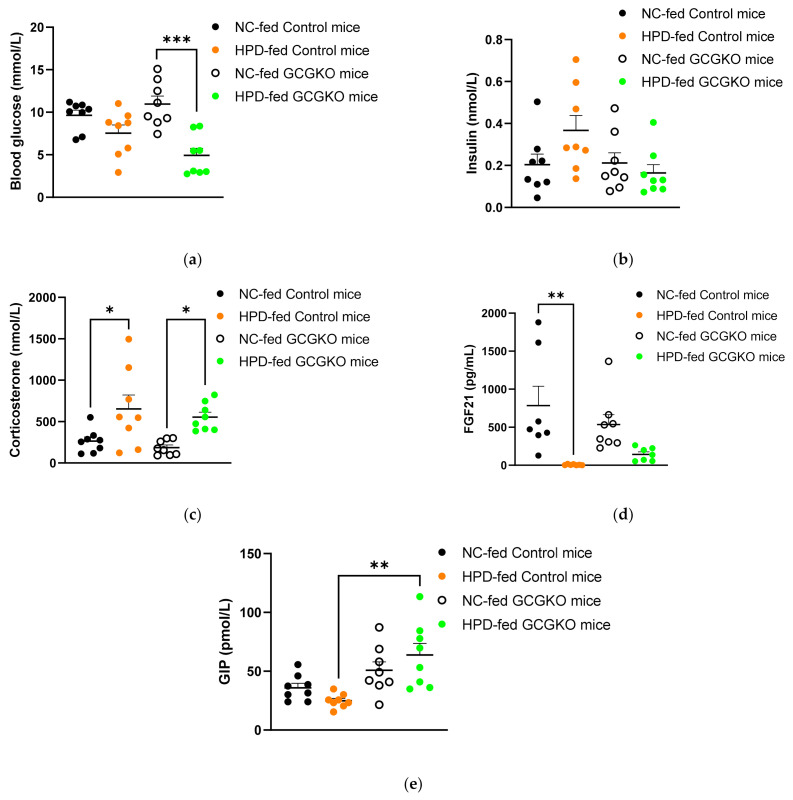
Blood glucose levels and hormonal response to HPD feeding in the presence and absence of proglucagon-derived peptides. (**a**) Blood glucose levels in NC-fed control mice (black dots; *n* = 8), HPD-fed control mice (orange dots; *n* = 8), NC-fed GCGKO mice (white dots; *n* = 8), and HPD-fed GCGKO mice (green dots; *n* = 8) under ad libitum feeding conditions after 1 week of feeding (*** *p* < 0.001). Plasma concentration of (**b**) insulin, (**c**) corticosterone, (**d**) FGF21, and (**e**) GIP in NC-fed control mice (black dots; *n* = 7–8), HPD-fed control mice (orange dots; *n* = 7–8), NC-fed GCGKO mice (white dots; *n* = 8), and HPD-fed GCGKO mice (green dots; *n* = 7–8) after 1 week of feeding (* *p* < 0.05, ** *p* < 0.01). Data are expressed as mean ± SEM.

HPD feeding resulted in clearly lower blood glucose levels (4.9 ± 0.8 mM) in the GCGKO mice (Figure 4a). Plasma insulin levels were not different among the four groups (Figure 4b). Plasma corticosterone levels were increased under HPD feeding both in the control mice and the GCGKO mice, and no significant difference was observed between HPD-fed control mice and GCGKO mice (Figure 4c). Plasma FGF21 levels were markedly suppressed by HPD feeding in the control mice. On the other hand, this decrease in plasma FGF21 levels by HPD feeding was partially attenuated in the GCGKO mice (Figure 4d). We previously reported that plasma GIP levels are increased in GCGKO mice [28], and plasma GIP levels in HPD-fed GCGKO mice were significantly higher than those in the HPD-fed control mice (Figure 4e). The HPD-fed control mice presented with lower ad libitum plasma GIP levels than NC-fed control mice (Figure 4e). However, the difference between NC- and HPD-fed controls was not significant in the statistical analyses involving all four groups (Figure 4e).

### 3.6. HPD Feeding Diminishes Triglyceride and Glycogen Content in the Liver, Regardless of the Presence or Absence of Proglucagon-Derived Peptides

**Figure 5:**
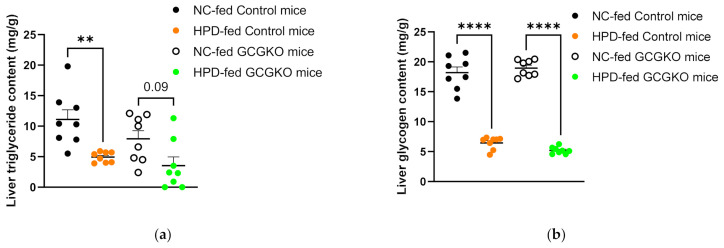
HPD feeding diminishes triglyceride and glycogen content in the liver, regardless of the presence of proglucagon-derived peptides. Liver (**a**) triglycerides and (**b**) glycogen levels in NC-fed control mice (black dots; *n* = 8), HPD-fed control mice (orange dots; *n* = 8), NC-fed GCGKO mice (white dots; *n* = 8), and HPD-fed GCGKO mice (green dots; *n* = 8) after 1 week of feeding (** *p* < 0.01, **** *p* < 0.0001). Data are expressed as mean ± SEM.

Although not statistically significant, the liver triglyceride content under NC feeding was lower in the GCGKO mice than in the control mice. HPD feeding significantly reduced triglyceride content in control mice. The HPD-induced decrease in triglyceride content was less pronounced in the GCGKO mice (Figure 5a). No significant difference in liver glycogen level was observed between the control and GCGKO mice. HPD feeding for 7 days markedly diminished the glycogen level in both groups of mice (Figure 5b). As shown in Figure 3c, no significant difference in liver weight was observed among the four groups.

### 3.7. GCG Is Required for Homeostasis of Amino Acid Metabolism in Response to Increased Protein Intake

**Figure 6:**
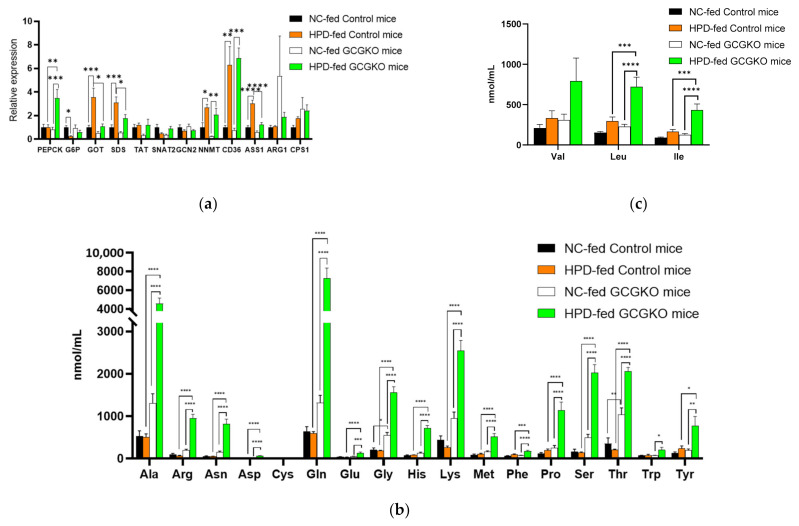
Glucagon is required for homeostasis of amino acid metabolism in response to increased protein intake. (**a**) mRNA expression levels of Pepck, G6pc, Got, Sds, Tat, Snat2, Gcn2, Nnmt, Cd36, ASS1, ARG1, and CPS1 in the liver in NC-fed control mice (black bars; *n* = 8), HPD-fed control mice (orange bars; *n* = 8), NC-fed GCGKO mice (white bars; *n* = 8), and HPD-fed GCGKO mice (green bars; *n* = 8) after 1 week of feeding (* *p* < 0.05, ** *p* < 0.01, *** *p* < 0.001). Plasma concentrations of (**b**) 17 amino acids and (**c**) 3 amino acids (BCAAs) in NC-fed control mice (black bars; *n* = 8), HPD-fed control mice (orange bars; *n* = 8), NC-fed GCGKO mice (white bars; *n* = 8), and HPD-fed GCGKO mice (green bars; *n* = 8) after 1 week of feeding (* *p* < 0.05, ** *p* < 0.01, *** *p* < 0.001, **** *p* < 0.0001). Data are expressed as mean ± SEM.

GCG plays a crucial role in amino acid metabolism in the liver, and animal models with defective GCG activity show hyperaminoacidemia [22]. To assess the impact of HPD feeding on metabolism in the presence and absence of proglucagon-derived peptides, gene expression in the liver was analyzed. Phosphoenolpyruvate carboxykinase (*Pepck*) and glucose-6-phosphate phosphatase (G6P) are regarded as rate-limiting enzymes of gluconeogenesis and are regulated by GCG [36]. However, the expression of *Pepck* and *G6pc* (the catalytic subunit of G6P) mRNAs was not induced in the control mice by HPD feeding for 7 days (Figure 6a). On the other hand, the *Pepck* mRNA was significantly increased in the GCGKO mice despite the absence of GCG, as was observed in the duodenum (Figure 3f).

We previously reported that the expression levels of soluble glutamate oxaloacetate transaminase (*Got*), serine dehydratase (*Sds*), tyrosine amino transferase (*Tat*) and argininosuccinate synthetase 1(*ASS1*) mRNAs were decreased in GCGKO mice [21], and similar results were observed in NC-fed control and GCGKO mice. The expression of *Got*, *Sds,* and *ASS1* mRNAs was strongly induced by HPD feeding in control mice and was significantly higher than in HPD-fed GCGKO mice, indicating that HPD feeding increased the expression of gene-encoding enzymes involved in amino acid metabolism and the urea cycle in a GCG-dependent manner. On the other hand, the expression of arginase 1 (*Arg1*) and carbamylphosphate synthetase 1 (*Cps1*) was not induced by HPD. These results suggest that not all enzymes involved in the urea cycle are regulated by GCG, and similar results were observed in GCG-blockade experiments using an anti-GCG receptor antibody [19].

Expression levels of *Slc38a2/Snat2* and *Gcn2/Eif2ak4* mRNA were also analyzed. SLC38A2 is involved in amino acid transport and is downregulated in GcgRKO [26]. GCN2/EIF2AK4 is involved in the regulation of gene expression in response to intracellular amino acids [37]. No significant difference in the expression of these genes was observed among the four groups. On the other hand, the expression levels of nicotinamide N-methyltransferase (*Nnmt*) and *Cd36* were significantly increased by HPD feeding in both the control and GCGKO mice, suggesting a regulatory mechanism independent of proglucagon-derived peptides (Figure 6a).

Concordant with our previous report [21], plasma concentrations of amino acids were markedly increased in GCGKO mice compared to the control mice under NC feeding (Figure 6b,c). Feeding GCGKO mice with HPD resulted in a marked increase in plasma amino acid levels (Figure 6b,c). The concentration of glutamine was 7.3 ± 1.1 mM, which was higher than the glucose concentration in HPD-fed GCGKO mice (4.9 ± 0.8 mM, Figure 4a). The difference between NC- and HPD-fed controls did not reach significance in the statistical analyses involving all four groups.

### 3.8. Response to Insulin Administration Was Attenuated in HPD-Fed GCGKO Mice

**Figure 7:**
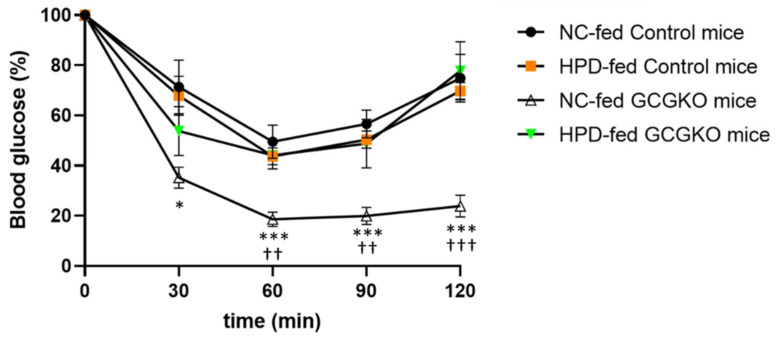
Response to insulin administration was attenuated in HPD-fed GCGKO mice. Percentage decrease in blood glucose levels during insulin tolerance test (ITT) in NC-fed control mice (black circles; *n* = 7), HPD-fed control mice (orange square; *n* = 8), NC-fed GCGKO mice (white triangle; *n* = 8), and HPD-fed GCGKO mice (green triangle; *n* = 7) after 1 week of feeding (* *p* < 0.05, *** *p* < 0.001, compared with NC-fed GCGKO mice and NC-fed control mice at the indicated time-points; ^††^  *p* < 0.01, ^†††^  *p* < 0.001, compared with NC-fed GCGKO mice and HPD-fed GCGKO mice at the indicated time-points). Data are expressed as mean ± SEM.

It has been reported that increased protein ingestion induces insulin resistance in humans [38,39,40,41]. On the other hand, GCGKO mice have been reported to be susceptible to hypoglycemia on insulin administration [20]. No significant difference in response to insulin administration was observed between NC- and HPD-fed control mice. However, insulin hypersensitivity in GCGKO mice fed NC was ameliorated in GCGKO mice fed HPD, although the underlying mechanism remains unclear (Figure 7).

## 4. Discussion

In the present study, we analyzed the metabolic impact of HPD feeding for a week in the presence and absence of proglucagon-derived peptides, including GCG and GLP-1. In both the GCGKO and control mice, caloric intake was reduced, resulting in body weight loss and a significant decrease in WAT weight. It has been reported that diets rich in protein potentiate GCG secretion in both mice and humans [6,7,8,9,10,11,12,42,43]. In addition, weight loss concomitant with a reduction in adipose tissue weight has been documented in rodent models fed an HPD [35,43,44,45]. Combining these data, GCG likely plays a role in body weight loss induced by HPD. Indeed, it has been reported that GCG is involved in the regulation of BAT activity and energy expenditure [46,47,48]. Therefore, we analyzed the expression levels of *Ucp1* and *Dio2* mRNA in BAT in WT mice as surrogate markers for energy expenditure (Figure 2e) [49]. Although plasma GCG levels were increased (Figure 1b), *Ucp1* and *Dio2* mRNA expression were suppressed in HPD-fed mice (Figure 2e). Therefore, GCG and thermogenesis in BAT appeared to be uncoupled in response to HPD feeding, and the contribution of proglucagon-derived peptides in weight loss should be limited. This result is consistent with data observed in mice fed for 12 weeks with an HPD (with 60% of their energy intake as protein) [43].

FGF21, which promotes energy expenditure, has been postulated as a mediator of GCG action [50], and regulatory mechanisms of FGF21 production by GCG have been explored [51]. However, production of FGF21 is also regulated by protein intake and is increased by low-protein diets [31,37]. In the present study, HPD feeding diminished plasma FGF21 levels in both the control and GCGKO mice. As HPD feeding significantly increased plasma GCG levels in WT mice (Figure 1b), the regulatory link between GCG and FGF21 appears to be uncoupled on HPD feeding. On the other hand, HPD-induced suppression of FGF21 was somewhat attenuated in GCGKO mice (Figure 4d), although the biological significance of this observation remains obscure.

Caloric intake decreased and protein intake increased to similar extents in control and GCGKO mice (Figure 3c). It has been reported that the expression of *Pepck* mRNA in the intestine is increased by HPD in rats, and the resulting increase in intestinal gluconeogenesis induces an anorectic action via vagus nerve activation [35]. On the other hand, the GCG receptor is reported to be expressed in the intestine and is possibly involved in intestinal gluconeogenesis [52]. In the present study, *Pepck* mRNA expression in the duodenum was increased in both the control and GCGKO mice. Therefore, proglucagon-derived peptides are not required to induce intestinal gluconeogenesis. The contribution of proglucagon-derived peptides in the HPD-induced decrease in food intake and weight loss appears to be negligible, although these peptides are regarded as anorectic peptides. Recently, it is reported that GABA_A_ receptor-α5 and the 5-HT_3_ receptor signaling in the melanocortin 4 receptor (MC4R) neurons located in the dorsal bed nucleus of the stria terminus participates in food consumption, food preference and body weight [53], and MC4R and 5-HT signaling in the dorsal raphe nucleus plays an important role in energy expenditure [54]. Whether this signaling contributes to the regulation of the food consumption and energy expenditure in mice fed HPD should be investigated in future studies.

The impact of the absence of proglucagon-derived peptides was marginal in the control of food intake, body weight, and energy expenditure, including FGF21 expression. On the other hand, homeostasis of amino acid metabolism was largely altered in GCGKO mice, especially when fed HPD. Changes in blood glucose levels and plasma amino acid levels by HPD feeding in the control mice were marginal (Figure 4a and Figure 6b,c). In statistical analyses involving all four groups, differences between the control and GCGKO mice were far greater than the difference between NC- and HPD-fed control mice. Therefore, the difference between NC- and HPD-fed control mice did not reach statistical significance. On the other hand, HPD feeding resulted in decreased blood glucose levels and markedly increased plasma amino acid levels in GCGKO mice (Figure 4a and Figure 6b,c). These results indicate that conversion of amino acids into glucose is severely attenuated in GCGKO mice in response to altered chronic protein intake, in line with the results of mice treated with glucagon receptor antibody and Gluc-DTR mice after acute intraperitoneal administration of mixed amino acids [26]. Whether long-term exposure to HPD induces glucagon resistance [26,55,56] should be investigated in future studies.

SDS deaminates serine and produces pyruvate and ammonia, and aspartate aminotransferase (AST/GOT) converts aspartate to oxaloacetate. The products of these enzymes, pyruvate and oxaloacetate, serve as substrates for gluconeogenesis. HPD feeding increased the expression of *Sds* and *Got* mRNAs in the control mice; however, such a response to HPD feeding was largely attenuated in GCGKO mice, accounting for the disrupted homeostasis of amino acid metabolism in response to increased protein intake (Figure 6a). While the increase in *Sds* and *Got* expression by HPD feeding was attenuated in GCGKO mice, *Nnmt* and *Cd36* expression were significantly increased in the HPD-fed GCGKO mice, similar to levels in HPD-fed control mice. Although the detailed mechanisms remain unclear, other hormonal signals such as corticosterone (Figure 4c) may be involved in the GCG-independent response to HPD feeding.

It has been reported that protein ingestion induces insulin resistance and that an increase in BCAA is involved in the underlying mechanism [38,39,40,41]. In the present study, no significant changes in blood glucose levels in response to insulin administration were observed in control mice after feeding with HPD for a week. On the other hand, HPD-fed GCGKO mice showed a significantly attenuated response compared to NC-fed GCGKO mice (Figure 7). As remarkable increases in plasma amino acids were observed in GCGKO mice, but not in control mice, the magnitude of the changes in amino acid metabolism may be linked to the differential response in blood glucose levels to insulin administration.

While the liver, the target organ of GCG, plays a major role in amino acid metabolism, the skeletal muscle also plays an important role in the metabolism of BCAAs. Meanwhile, BCAAs promote protein synthesis in the muscle [57,58]. In the present study, reduced caloric intake and increased protein intake did not result in a significant change in skeletal muscle weight per body weight. Increased BCAA levels in plasma may protect against loss of muscle under reduced caloric intake (Figure 3c and Figure 6c).

The limitation of the current study is that it is difficult to distinguish the effects of increased protein intake from those of decreased carbohydrate intake, as these changes are inevitably coupled. Indeed, the increase in corticosterone (Figure 4b) may be attributable to the decrease in carbohydrate intake rather than the increase in protein intake. Nevertheless, it is reasonable that changes in plasma amino acid levels and genes involved in amino acid catabolism are mainly induced by changes in protein intake.

## 5. Conclusions

In the present study, we analyzed the pleiotropic effects of HPD feeding in the presence and absence of proglucagon-derived peptides. Results showed that the impact of proglucagon-derived peptides on intestinal gluconeogenesis, energy expenditure, and control of food intake and body weight is limited. On the other hand, hyperaminoacidemia was markedly aggravated in the mice deficient in proglucagon-derived peptides, underscoring the importance of glucagon in the homeostasis of plasma amino acid levels in response to altered protein intake.

## Figures and Tables

**Table 1:** Primers used for quantitative real-time PCR (qPCR).

Gene	Forward Primers (5′–3′)	Reverse Primers (5′–3′)
Ucp1	GGGCCCTTGTAAACAACAAA	GTCGGTCCTTCCTTGGTGTA
Dio2	GCTTCCTCCTAGATGCCTAC	TGGCTGAACCAAAGTTGACC
Pepck	GTGTTTGTAGGAGCAGCCATGAG	TAGCCGAAGAAGGGTCGCAT
G6p	CGGATCTACCTTGCTGCTCA	AACAAGAAGATGGTGATGAGACAAT
Got	TTGGTCTCACATCACTGAGCA	GATGGAGGTAGCGACGTAATCTAG
Sds	CAGCTTCCATGCTGCCATCAAG	CCTCCTGGTCTGAGATGACCTC
Tat	CCGAGCCATTGTGGACAACAT	GTTGACCACGAGACAAGCTGTTT
Snat2	TAATCTGAGCAATGCGATTGTGG	AGATGGACGGAGTATAGCGAAAA
Gcn2	CCCGGACATACTCCTCAGGAA	GGCTACCCACAGAGAAATGGA
Nnmt	TTTGACTGGTCCCCAGTGGT	GGCACAGCGTGCTGAGCAAG
Cd36	AGATGACGTGGCAAAGAACAG	CCTTGGCTAGATAACGAACTCTG
Ass1	GCGACTATGAGCCCATCGAC	GGCCCGCTCCTCTTTGTCAG
Arg1	TTCTGGGAGGCCTATCTTACAGA	CCACTGCCGTGTTCACAGTA
Cps1	GGAGTGGATACAAGAATGCTGAC	GCAGGCGGATGACATTGTTTTT
β-actin	CATCCGTAAAGACCTCTATGCCAAC	ATGGAGCCACCGATCCACA

## Data Availability

The data used to support the findings of this study are available from Y.S. (Yusuke Seino) upon request.
